# Toward a better definition of *EPCAM* deletions in Lynch Syndrome: Report of new variants in Italy and the associated molecular phenotype

**DOI:** 10.1002/mgg3.587

**Published:** 2019-03-27

**Authors:** Giulia Cini, Michele Quaia, Vincenzo Canzonieri, Mara Fornasarig, Roberta Maestro, Alberto Morabito, Angela Valentina D'Elia, Emanuele Damiano Urso, Isabella Mammi, Alessandra Viel

**Affiliations:** ^1^ Functional Oncogenomics and Genetics Centro di Riferimento Oncologico di Aviano (CRO), IRCCS Aviano PN Italy; ^2^ Pathology Centro di Riferimento Oncologico di Aviano (CRO), IRCCS Aviano PN Italy; ^3^ Gastroenterology Centro di Riferimento Oncologico di Aviano (CRO), IRCCS Aviano PN Italy; ^4^ Oncology Cittadella PD Italy; ^5^ Department of Medical Area University of Udine Udine Italy; ^6^ Department of Surgical Oncology and Gastroenterology University of Padua Padova Italy; ^7^ Medical Genetics Dolo Hospital Dolo VE Italy

**Keywords:** deletion, *EPCAM*, epigenetics, Lynch Syndrome, methylation, *MSH2*

## Abstract

**Background:**

Inherited epimutations of Mismatch Repair (MMR) genes are responsible for Lynch Syndrome (LS) in a small, but well defined, subset of patients. Methylation of the *MSH2* promoter consequent to the deletion of the upstream *EPCAM* gene is found in about 1%–3% of the LS patients and represents a classical secondary, constitutional and tissue‐specific epimutation. Several different *EPCAM* deletions have been reported worldwide, for the most part representing private variants caused by an *Alu*‐mediated recombination.

**Methods:**

712 patients with suspected LS were tested for MMR mutation in our Institute. *EPCAM* deletions were detected by multiplex ligation‐dependent probe amplification (MLPA) and then defined by Long‐Range polymerase chain reaction (PCR)/Sanger sequencing. A comprehensive molecular characterization of colorectal cancer (CRC) tissues was carried out by immunohistochemistry of MMR proteins, Microsatellite Instability (MSI) assay, methylation specific MLPA and transcript analyses. In addition, somatic deletions and/or variants were investigated by MLPA and next generation sequencing (NGS).

**Results:**

An *EPCAM* deletion was found in five unrelated probands in Italy: variants c.556‐490_*8438del and c.858+1193_*5826del are novel; c.859‐1430_*2033del and c.859‐670_*530del were previously reported. All probands were affected by CRC at young age; tumors showed MSI and abnormal MSH2/MSH6 proteins expression. *MSH2* promoter methylation, as well as aberrant in‐frame or out‐of‐frame *EPCAM/MSH2* fusion transcripts, were detected in CRCs and normal mucosae.

**Conclusion:**

An *EPCAM* deletion was the causative variant in about 2% of our institutional series of 224 LS patients, consistent with previously estimated frequencies. Early age and multiple CRCs was the main clinical feature of this subset of patients.

## 1 INTRODUCTION

Pathogenic variants in *MLH1, MSH2, MSH6 *and *PMS2* Mismatch Repair (MMR) genes (OMIM: 120436, 609309, 600678 and 600259) are causative of Lynch Syndrome (LS), an autosomal dominant condition that confers elevated risk of developing colorectal cancer (CRC), endometrial cancer (EC) and several other types of cancer. Typical phenotypic features of LS tumors are Microsatellite Instability (MSI) and loss of MMR protein expression (Lynch et al., 2009).

Biallelic inactivation of one of MMR genes, as a result of combined germline and second hit somatic mutations, facilitates carcinogenesis. Nonsense, missense, frameshift and splicing variants, as well as deletions of one or more exons, are responsible for loss of function of the inherited, predisposing MMR allele. In addition, some so‐called epimutations are reported. Epimutation is referred as a heritable change that does not alter the DNA sequence but affects gene expression via DNA methylation or histone modification. In LS, constitutional epimutations are mainly reported for the *MLH1 *gene*,* either as primary event arisen de novo in gametogenesis or as secondary event, caused by an *in cis* heritable genetic variant (Cini et al., 2015; Hitchins, 2013).

As about the *MSH2* gene, a constitutional tissue‐specific promoter methylation can be inherited as secondary epimutation, due to a deletion involving the last exons of the *EPCAM* gene (OMIM: 185535) that maps upstream *MSH2*. As a consequence, *EPCAM* is brought close to *MSH2* and, in EpCAM expressing tissues, a read‐through *EPCAM/MSH2* fusion transcript is generated while the native *MSH2* promoter is hypermethylated and silenced (Kovacs, Papp, Szentirmay, Otto, & Olah, 2009; Ligtenberg et al., 2009). This complex event represents the first hit in a well‐defined subset of LS patients (1%–3% of the total) (Kuiper et al., 2011) that are typified by the expression of aberrant *EPCAM/MSH2* fusion transcripts in normal and tumor colon tissues (Kovacs et al., 2009; Ligtenberg et al., 2009).

Several different *EPCAM* deletions have been described worldwide, in some cases as recurrent/founder mutations (Dymerska et al., 2017; Eguchi et al., 2016; Mur et al., 2014; Spaepen et al., 2013). It has been established that the mechanism underlying these rearrangements is an *Alu*‐mediated deletion, involving different highly homologous *Alu* sequences that are interspersed in intronic and intergenic regions within the *EPCAM/MSH2* locus (Tutlewska, Lubinski, & Kurzawski, 2013).

Here we report on the identification and characterization of four different *EPCAM* deletions in five unrelated Italian LS families, and their epigenetic effect on the *MSH2 *locus.

## 2 MATERIALS AND METHODS

From 1995 to 2017, DNA from a total of 712 unrelated cancer patients was collected in a diagnostic setting by the Functional Oncogenetics and Oncogenomics Laboratory at the CRO National Cancer Institute (Aviano, Italy). Patients were enrolled for genetic testing because of personal and family history consistent with LS and/or evidence of high MSI (MSI‐H) and/or negative for MMR protein according to immunohistochemistry (IHC). Diagnosis of LS was confirmed by MMR and *EPCAM* genetic testing in 224/712 patients.

The genetic testing protocol and use of DNA samples for research purposes was approved by the Local Independent Ethics Committee (CRO‐15‐1997). Written informed consent was obtained from all participants of the present study.

Mismatch Repair protein IHC was performed on Formalin‐Fixed and Paraffin‐Embedded (FFPE) sections, stained on the Ventana BenchMark Ultra platform (Ventana Medical Systems, Inc., Oro Valley AZ). MLH‐1 (M1), MSH2 (G219‐1129), MSH6 (44) and PMS2 (EPR3947) (Roche Diagnostics, Indianapolis, IN) monoclonal antibodies were used to qualitatively identify human MMR proteins. Lesions were considered negative for protein expression when a complete absence of nuclear staining was evident in tumor cells with concomitant nuclear staining of adjacent normal epithelial and stromal cells.

Genetic screening for MMR genes was carried out by Sanger sequencing of coding sequences (*MSH2*: NM_000251.2, *MLH1*: NM_000249.3, *MSH6*: NM_000179.2) and with SALSA® multiplex ligation‐dependent probe amplification (MLPA)® probemixes P003 (MLH1/MSH2), P072 (MSH6) and P248 (MLH1/MSH2 confirmation), followed by copy number variation analysis with Coffalyser.net (MRC‐Holland, Amsterdam, Holland).

Long‐Range polymerase chain reactions (PCRs) for breakpoints detection were performed with Expand™ Long Template PCR System (Merck KGaA, Darmstadt, Germany). Shorter PCRs including breakpoints were obtained with GoTaq (Promega, Madison, WI) (primers and conditions available upon request). Polymerase chain reaction products were purified from agarose gel with the Wizard® SV Gel and PCR Clean‐Up System (Promega), then sequenced using a modified BigDye® cycle sequencing protocol (30x 94°C 30″, 55°C 30″, 60°C 2′), to prevent secondary structures due to *Alu*‐mediated hybridization.

Tumor and normal mucosa methylation statuses were assessed using Methylation Specific‐MLPA (MS‐MLPA) with SALSA® MLPA® ME011 MMR genes probemix (MRC‐Holland). Blood DNA was used as a control.

Mutation analysis of tumor DNA was performed on the two available frozen CRC samples with a TruSeq Custom Amplicon (Illumina, San Diego, CA) next generation sequencing (NGS) panel, including coding sequences of *MSH2*, *MSH6* and *MLH1* genes. Libraries were run on an Illumina MiSeq platform, with a mean coverage >4,000 reads. MLPA analysis for loss of heterozygosity (LOH) detection was carried out on frozen and FFPE tumor samples with the SALSA® MLPA® probemix P072‐C1 (MSH6).

Aberrant transcripts were investigated in RNAs extracted from frozen or FFPE samples. In brief, RNA from tumor and/or normal mucosa of carriers was purified with the TRIzol™ Reagent (ThermoFisher Scientific, Waltham, MA) or the RNeasy FFPE kit (Qiagen, Hilden, Germany) according to manufacturer's protocols, and cDNA was retrotranscribed using the SuperScript™ III Reverse Transcriptase (ThermoFisher Scientific) and random primers. The presence of fusion transcripts was evaluated by amplification and direct sequencing of PCRs covering different regions between exon 4 or 7 of the *EPCAM* gene and exon 1 or 2 or 3 of *MSH2* (primers and details upon request). The reference sequences NM_002354.2 and NG_012352.2 (*EPCAM*) and NM_000251.2 and NG_007110.2 (*MSH2*) were used for reporting aberrant transcripts and genomic deletions.

## 3 RESULTS

Of the 224 patients tested positive for LS in our laboratory from 1995 to 2017, five unrelated patients, all Italians, carried a partial deletion of the *EPCAM* gene, involving exons 6‐9 (families AV114 and UD18) or exons 8‐9 (families PD31, AV182 and PD78), as assessed by MLPA analyses. No other pathogenic variants were found in *MSH2* and *MSH6* genes of these patients.

**Figure 1:**
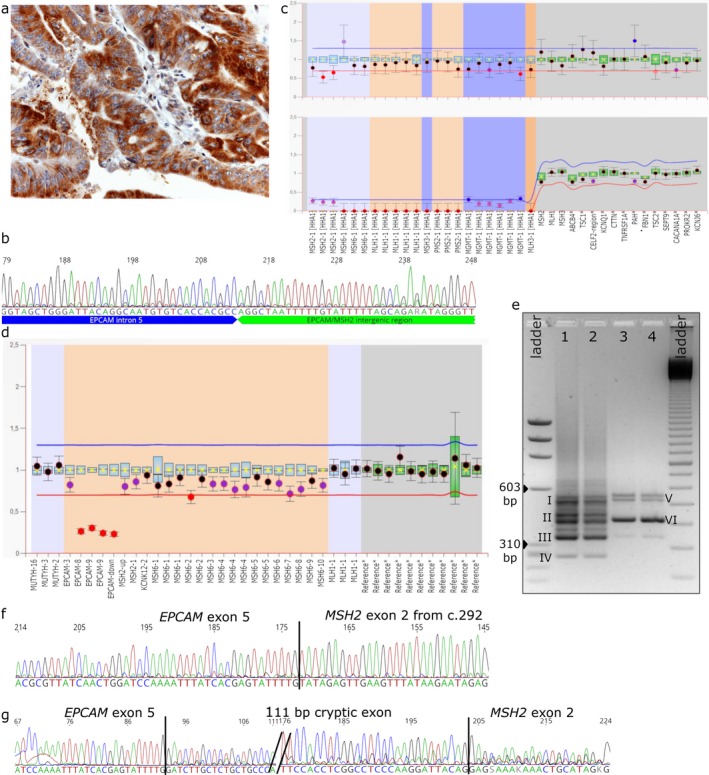
Representative molecular characterization of patient CFS396 carrying the *EPCAM Del_16.5*. (a) Immunohistochemistry showing that MSH2 protein is exclusively expressed in the cytoplasm of the tumor cells (H&E counterstain, O.M. 400x). (b) Sanger sequence of breakpoint. (c) Coffalyser analysis of methylation specific‐MLPA in tumor DNA, displaying *MSH2* promoter methylation. (d) Coffalyser analysis of MLPA in tumor DNA, in which partial loss of heterozygosity of *EPCAM* is evidenced, due to a large somatic deletion involving also *MSH2* and *MSH6 *genes. (e) Agarose‐gel electrophoresis showing aberrant *EPCAM/MSH2* fusion transcripts amplified from tumor (lane 1–2) and normal mucosa (lanes 3–4) cDNAs; DNA ladders: ΦX174 DNA‐Hae III Digest (left) and 100 bp ladder (right). Roman numerals indicate the main PCR products that were sequenced. (f) Sanger sequence of an in‐frame fusion transcript (corresponding to amplicon III). (g) Sanger sequence of an out‐of‐frame fusion transcript (amplicon I and V)

All probands were diagnosed with MSI‐H CRC at young age (34–50 years), and had a family history of CRC. Immunohistochemistry, performed on a representative tumor of the proband, revealed abnormal MSH2 and MSH6 expression (Table 1). Notably, besides tumors with clear‐cut loss of nuclear MSH2/MSH6 staining, one tumor retained focal MSH2 expression and some showed heterogeneous MSH2 and/or MSH6 cytoplasmic staining (Figure 1a).

**Table 1:** *EPCAM* deletion carriers

Family ID	Patient ID	Sex	Clinical phenotype	IHC MSH2/MSH6	MSI	*MSH2* methylation	Somatic LOH
PD31	CFS394	F	CRC 45 y^b^, 55 y		H		
	CFS395^a^	M	CRC 43, 68 y^b^, duodenum 62 y, larynx^c^ 67 y	−/Cytoplasmic	H	33%(N) 23%(T)	No
	CFS890	M	Healthy 37 y				
	CFS892	F	Healthy 39 y				
	CFS918	F	CRC 32 y, breast^c^ 47 y				
	CFS919	F	Healthy 44 y				
	CFS920	M	Healthy 57 y				
AV114	CFS396^a^	F	CRC 36, 54 y^b^, 54 y	Cytoplasmic/cytoplasmic	H	29%(N) 50%(T)	*EPCAM‐MSH2‐MSH6* loss
	CFS487	M	CRC 42 y^b^	Cytoplasmic/cytoplasmic	H	Not evaluable	*EPCAM‐MSH2‐MSH6* loss
	CFS488	F	Healthy 35 y				
	CFS516	M	CRC 25 y^b^		H		
UD18	CFS825^a^	M	CRC 39, 53 y^b^	−/−	H	23%(T)	No
	CFS913	M	Healthy 19 y				
	CFS914	M	Healthy 21 y				
AV182	CFS1043^a^	M	CRC 50, 50, 63 y^b^	−/−	H	23%(T)	*EPCAM* loss
PD78	CFS1475^a^	M	CRC 34 y^b^	Focal/+	H	23%(T)	No
	CFS1541	M	Healthy 39 y				

Note

CRC, colorectal cancer; H, MSI‐H; IHC, immunoistochemistry; LOH, loss of heterozygosity; LS, Lynch Syndrome; MSI, microsatellite instability; N, normal mucosa; T, tumor; y, years (age of tumor onset, if affected, and age at registration, if healthy).

a Probands.

b Tumors tested.

c Tumors not included in LS spectrum.

Twenty relatives from four families were tested for *EPCAM* deletion: eight healthy subjects (age 19–57 years) and four patients affected by early‐onset CRC (age 25–45 years) tested positive at MLPA for the deletion detected in the proband. Clinical and molecular data of all carriers are listed in Table 1.

Long range PCRs from carriers produced smaller amplicons compared with wild‐type controls. Characterization of the breakpoints (Figure S1) revealed four different deletions, *Del_4.9, Del_16.5 *(Figure 1b)*, Del_2.6, Del_11.5* (Table 2), all residing in repetitive sequences and involving two *Alu* regions with very high homology (79%–88%); a microhomology sequence (from 9 to 36 bp) was identified for all the described deletions. Families AV114 and UD18 carried the same deletion.

**Table 2:** Summary of *EPCAM* deletions

Family ID	Deletion ID	Deleted *EPCAM* exons	Annotation^a^	Distance from *MSH2* (kb)	Size (bp)	*Alu* family	Microhomology region (bp)	Max sequence homology (%)
PD31	*Del_4.9*	8‐9	c.859–1430_*2033del	13	4,909	Sx/Sq2	14	84% for 211/250
AV114 UD18	*Del_16.5*	6‐9	c.556–490_*8438del	7	16,588	Sp/Sx	9	82% for 196/239
AV182	*Del_2.6*	8‐9	c.859–670_*530del	15	2,648	Sx1/Sx4	19	79% for 246/312
PD78	*Del_11.5*	8‐9	c.858 + 1193_*5826del	10	11,492	Sp/Sp	36	88% for 260/296

a *EPCAM* reference sequence NM_002354.2.

Mismatch Repair gene methylation status was ascertained in five tumors (CFS395T, CFS396T, CFS825T, CFS1043T, CFS1475T) and two normal mucosae (CFS395N and CFS396N). *MSH2* promoter was methylated, at different percentages (23%–50%), in both normal and tumor tissues (Table 1 and Figure 1c).

No pathogenic variant acting as a second hit was found by NGS screening in either *MSH2* or other MMR genes in the DNA from CFS395T and CFS396T tumors. MLPA performed on available tumor samples highlighted a variegate pattern ranging from retention of the second *EPCAM* allele to LOH of various extent and amplitude (up to *MSH2* and *MSH6* loci) (Table 1 and Figure 1d).

Several *EPCAM‐MSH2* fusion transcripts were detected by PCR in normal mucosa and in CRC samples CFS395T, CFS396T and CFS1475T (Table S1 and Figure 1e), but not in wild‐type controls (not shown).

Sequencing of PCR products evidenced at least five different aberrant transcripts originated from *Del_16.5*: three in‐frame and two creating a frameshift with a premature stop codon in exon 2 (Figure 1f‐g shows a representative case). Also *Del_4.9* and *Del_11.5 *yielded different in‐frame and out‐of‐frame *EPCAM/MSH2* fusion transcripts (Table 1).

## 4 DISCUSSION

An increasing body of evidence supports the relevance of epigenetic modifications in the pathogenesis of hereditary syndromes. The elucidation of the primary event causing the epigenetic change is crucial to ascertain the actual secondary nature of the epimutation and to optimize cascade testing in family members. In the context of LS testing, the importance of epimutations analysis is universally recognized and *EPCAM* deletion is a well‐known primary event. In the last 10 years many different variants of this gene have been described; however, construction of a complete and curated public database for *EPCAM* deletions is still in progress (https://databases.lovd.nl/shared/genes/EPCAM), and the actual frequency of *EPCAM* mutations in LS is poorly defined. In particular, no precise estimate of *EPCAM* variant incidence in the Italian LS population is available so far. Our institutional database includes 224 molecularly confirmed LS unrelated families, five of which with *EPCAM* deletion (>2%), a frequency consistent with literature data (Tutlewska et al., 2013).

All *EPCAM* deletions reported worldwide are *bona fide* A*lu *recombination‐mediated variants, as they are the four deletions presented here. With the exception of few founder mutations identified in the Netherlands and Poland (Dymerska et al., 2017; Niessen et al., 2009), in general deletions in the *EPCAM* gene are rare, sometimes private, variants with no recombination hotspot sites. Two of the four deletions (*Del_16.5* and *Del_11.5*) that we describe here are novel, not recorded in any databases or literature. The *Del_2.6* variant was identical to that reported in the Netherlands (Kuiper et al., 2011). The *Del_4.9* variant was first described as founder mutation in Dutch population and in the large American Family R (Kuiper et al., 2011; Ligtenberg et al., 2009; Lynch et al., 2011; Niessen et al., 2009). Interestingly, the *Del_4.9* variant described here and that previously reported (Ligtenberg et al., 2009) (GenBank: FJ347525.1) had different breakpoints, although involving the same *Alu* sequences, and our family had no known Dutch ancestry.

For three out of four deletions, we had evidence in both normal and tumor colon tissues, of *EPCAM/MSH2* fusion transcripts. These were concomitant to *MSH2* promoter methylation, which acted as a first hit for gene inactivation.

In all *EPCAM* deleted tumors analyzed, MSH2/MSH6 IHC nuclear expression was always abnormal. In three cases the loss of nuclear MSH2 staining was compatible with a large somatic deletion of the locus. A peculiarity was the MSH2 cytoplasmic staining detected in two cases. This aberrant localization is similar to that described in a previous report of a larger deletion involving both *EPCAM* and the first two exons of *MSH2* (Sekine et al., 2017), and is consistent with the production of the alternative in‐frame fusion transcripts, predicted to be translated into chimeric proteins. It is tempting to speculate that the fusion with EPCAM alters the intrinsic capability of MSH2 to form a functional complex with MSH6, which is responsible for MSH2/MSH6 nuclear shuttling (Gassman et al., 2011).

Somatic *EPCAM/MMR* second hits could be identified only in three out of six *EPCAM* deletion‐associated tumors. The sub‐optimal quality of FFPE DNA from samples CFS825T and CFS1475T prevented NGS testing, so somatic *MSH2* point‐mutations cannot be excluded. Instead, MSH2 deficiency remains unexplained in sample CFS395T, in which both MLPA and NGS analyses were performed, but neither LOH nor DNA sequence variants were detected.

*EPCAM* deletion carriers typically show high penetrance of early onset CRC and a CRC cumulative risk up to 75%; compared to *MSH2* mutation carriers rarely develop multiple tumors of different histotypes. Moreover, the cumulative risk of EC in *EPCAM* deletion carriers is lower (12% vs. 51%), although this risk is slightly increased for deletions more proximal to the *MSH2 *locus (Kempers et al., 2011). According to previous genotype‐phenotype correlations, even in our small dataset EC is absent and the most frequent tumor is CRC, with 16 CRCs in eight affected carriers. Notably, one duodenal tumor was also recorded, confirming a previous suggestion of increased risk (Kempers et al., 2011), but the data are insufficient for correlating this clinical phenotype to particular *EPCAM* genotypes. Given the presence of multiple metachronous CRCs, the gastro‐intestinal prevention protocols for *EPCAM* deletion carriers should be focused on colon‐rectum, but surveillance of small intestine is also warranted. It is worth noting that EC occurrence, even if rare, cannot be excluded, and the gynecologic surveillance is still advised for all women with an *EPCAM* deletion.

This report underscores the importance of *EPCAM* genetic screening in LS patients, especially in the presence of an atypical pattern of MSH2 protein expression in tumors. In perspective, a thorough molecular characterization of the breakpoints might help in genetic counseling by laying down the basis for improved tumor risk estimates.

## CONFLICT OF INTEREST

None declared.

## References

[mgg3587-bib-0001] Cini, G. , Carnevali, I. , Quaia, M. , Chiaravalli, A. M. , Sala, P. , Giacomini, E. , … Viel, A. (2015). Concomitant mutation and epimutation of the MLH1 gene in a Lynch syndrome family. Carcinogenesis, 36(4), 452–458. 10.1093/carcin/bgv015 25742745

[mgg3587-bib-0002] Dymerska, D. , Gołębiewska, K. , Kuświk, M. , Rudnicka, H. , Scott, R. J. , & Billings, R. , … Kurzawski, G. (2017). New EPCAM founder deletion in Polish population. Clinical Genetics, 92(6), 649–653. 10.1111/cge.13026 28369810

[mgg3587-bib-0003] Eguchi, H. , Kumamoto, K. , Suzuki, O. , Kohda, M. , Tada, Y. , Okazaki, Y. , & Ishida, H. (2016). Identification of a Japanese Lynch syndrome patient with large deletion in the 3’ region of the EPCAM gene. Japanese Journal of Clinical Oncology, 46(2), 178–184. 10.1093/jjco/hyv172 26613680

[mgg3587-bib-0004] Gassman, N. R. , Clodfelter, J. E. , McCauley, A. K. , Bonin, K. , Salsbury, F. R. , & Scarpinato, K. D. (2011). Cooperative nuclear localization sequences lend a novel role to the N‐terminal region of MSH6. PLoS ONE, 6(3), e17907 10.1371/journal.pone.0017907 21437237 PMC3060103

[mgg3587-bib-0005] Hitchins, M. P. (2013). The role of epigenetics in Lynch syndrome. Familial Cancer, 12(2), 189–205. 10.1007/s10689-013-9613-3 23462881

[mgg3587-bib-0006] Kempers, M. J. E. , Kuiper, R. P. , Ockeloen, C. W. , Chappuis, P. O. , Hutter, P. , Rahner, N. , … Ligtenberg, M. J. L. (2011). Risk of colorectal and endometrial cancers in EPCAM deletion‐positive Lynch syndrome: A cohort study. The Lancet Oncology, 12(1), 49–55. 10.1016/S1470-2045(10)70265-5 21145788 PMC3670774

[mgg3587-bib-0007] Kovacs, M. E. , Papp, J. , Szentirmay, Z. , Otto, S. , & Olah, E. (2009). Deletions removing the last exon of TACSTD1 constitute a distinct class of mutations predisposing to Lynch syndrome. Human Mutation, 30(2), 197–203. 10.1002/humu.20942 19177550

[mgg3587-bib-0008] Kuiper, R. P. , Vissers, L. E. L. M. , Venkatachalam, R. , Bodmer, D. , Hoenselaar, E. , Goossens, M. , … Ligtenberg, M. J. L. (2011). Recurrence and variability of germline EPCAM deletions in Lynch syndrome. Human Mutation, 32(4), 407–414. 10.1002/humu.21446 21309036

[mgg3587-bib-0009] Ligtenberg, M. J. L. , Kuiper, R. P. , Chan, T. L. , Goossens, M. , Hebeda, K. M. , Voorendt, M. , … Hoogerbrugge, N. (2009). Heritable somatic methylation and inactivation of MSH2 in families with Lynch syndrome due to deletion of the 3’ exons of TACSTD1. Nature Genetics, 41(1), 112–117. 10.1038/ng.283 19098912

[mgg3587-bib-0010] Lynch, H. T. , Lynch, P. M. , Lanspa, S. J. , Snyder, C. L. , Lynch, J. F. , & Boland, C. R. (2009). Review of the Lynch syndrome: History, molecular genetics, screening, differential diagnosis, and medicolegal ramifications. Clinical Genetics, 76(1), 1–18. 10.1111/j.1399-0004.2009.01230.x PMC2846640 19659756

[mgg3587-bib-0011] Lynch, H. T. , Riegert‐Johnson, D. L. , Snyder, C. , Lynch, J. F. , Hagenkord, J. , Boland, C. R. , … Ligtenberg, M. J. L. (2011). Lynch syndrome‐associated extracolonic tumors are rare in two extended families with the same *EPCAM* deletion. The American Journal of Gastroenterology, 106(10), 1829–1836. 10.1038/ajg.2011.203 21769135 PMC3805505

[mgg3587-bib-0012] Mur, P. , Pineda, M. , Romero, A. , del Valle, J. , Borràs, E. , Canal, A. , … Capellá, G. (2014). Identification of a founder EPCAM deletion in Spanish Lynch syndrome families. Clinical Genetics, 85(3), 260–266. 10.1111/cge.12152 23530899

[mgg3587-bib-0013] Niessen, R. C. , Hofstra, R. M. W. , Westers, H. , Ligtenberg, M. J. L. , Kooi, K. , Jager, P. O. J. , … Sijmons, R. H. (2009). Germline hypermethylation of MLH1 and EPCAM deletions are a frequent cause of Lynch syndrome. Genes, Chromosomes & Cancer, 48(8), 737–744. 10.1002/gcc.20678 19455606

[mgg3587-bib-0014] Sekine, S. , Ogawa, R. , Saito, S. , Ushiama, M. , Shida, D. , Nakajima, T. , … Sugano, K. (2017). Cytoplasmic MSH2 immunoreactivity in a patient with Lynch syndrome with an EPCAM–MSH2 fusion. Histopathology, 70(4), 664–669. 10.1111/his.13104 27896849

[mgg3587-bib-0015] Spaepen, M. , Neven, E. , Sagaert, X. , De Hertogh, G. , Beert, E. , Wimmer, K. , … Brems, H. (2013). EPCAM germline and somatic rearrangements in lynch syndrome: Identification of a novel 3′EPCAM deletion. Genes, Chromosomes and Cancer, 52(9), 845–854. 10.1002/gcc.22080 23801599

[mgg3587-bib-0016] Tutlewska, K. , Lubinski, J. , & Kurzawski, G. (2013). Germline deletions in the EPCAM gene as a cause of Lynch syndrome – literature review. Hereditary Cancer in Clinical Practice, 11(1), 9 10.1186/1897-4287-11-9 23938213 PMC3765447

