# Supplementary material for: Toward a better definition of EPCAM deletions in Lynch Syndrome: Report of new variants in Italy and the associated molecular phenotype
Source: Mol Genet Genomic Med. 2019 Mar 27;7(5):e587. doi: 10.1002/mgg3.587 (PMC6503020; doi:10.1002/mgg3.587)
Supplement: Supplementary file 1 [file MGG3-7-e587-s001.docx]

Supplementary Figure S1. Sanger sequences of amplicons including breakpoints for Del_4.9 (A), Del_16.5 (B), Del_2.6 (C) and Del_11.5 (D)


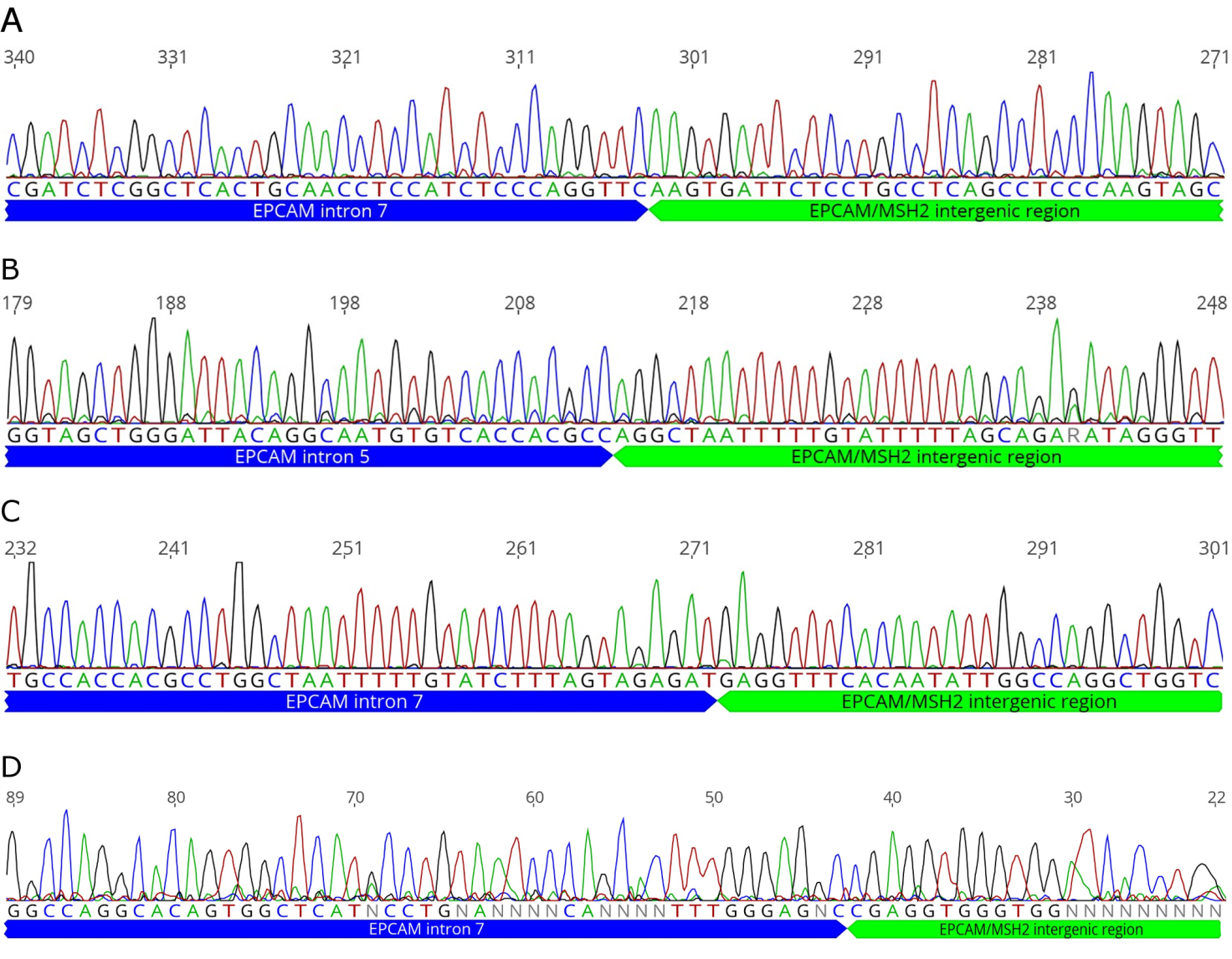


Supplementary Table S1. Fusion transcripts detected in tumors of the *EPCAM* deletion carriers

| Deletion ID | Sample | Fusion transcripts | Annotation^§^ | In-frame/out-of-frame |
| --- | --- | --- | --- | --- |
| *Del_4.9*  *Del_11.5* | **CFS395T**  **CFS1475T** | *EPCAM* exon 7/*MSH2* exon 2 | r.859_945delinsNM_000251.2:r.211_3209 | Out-of-frame |
|  |  | *EPCAM* exon 7/cryptic exon/*MSH2* exon 2 | r.859_945delinsNG_007110.2:g.833_901,insNM_000251.2:r.211_3209 | Out-of-frame |
|  |  | *EPCAM* exon 7/*MSH2* exon 2 (from c.292; p.98) | r.859_945delinsNM_000251.2:r.292_3209 | In-frame |
| *Del_16.5* | **CFS396T** | *EPCAM* exon 5/*MSH2* exon 2 (II, VI)* | r.556_945delinsNM_000251.2:r.211_3209 | Out-of-frame |
|  |  | *EPCAM* exon 5/cryptic exon/*MSH2* exon 2 (I, V)* | r.556_945delinsNG_007110.2:g.833_901,insNM_000251.2:r.211_3209 | Out-of-frame |
|  |  | *EPCAM* exon 5/*MSH2* exon 1 (from c.73; p.25) (I, V)* | r.556_945delinsNM_000251.2:r.73_3209 | In-frame |
|  |  | *EPCAM* exon 5/*MSH2* exon 2 (from c.292; p.98) (III)* | r.556_945delinsNM_000251.2:r.292_3209 | In-frame |
|  |  | *EPCAM* exon 5/*MSH2* exon 3 (IV)* | r.556_945delinsNM_000251.2:r.367_3209 | In-frame |

* Roman numerals in parentheses refer to the amplicons shown in figure 1E; ^§.^ *EPCAM* reference sequences: NM_002354.2 and NG_012352.2; *MSH2* reference sequence: NM_000251.2 and NG_007110.2
